# Differential impact of microcystins MC-LR and [D-Leu^1^]MC-LR in different areas of the rat brain after chronic exposure: oxidative stress and antioxidant responses

**DOI:** 10.1016/j.crmicr.2025.100401

**Published:** 2025-05-10

**Authors:** Hernando Marcelo, Cogo Pagella Joaquín, de la Rosa Florencia, Giannuzzi Leda, Cervino Claudio

**Affiliations:** a Instituto de Ciencias Básicas y Experimentales, Universidad de Morón, General Machado 914, Morón 1708, Argentina; b Department of Radiobiology, National Atomic Energy Commission, San Martin 1650, Argentina; c Red de Investigación de Estresores Marinos-Costeros en América Latina y el Caribe (REMARCO), Mar del Plata 7602, Argentina; d Consejo Nacional de Investigaciones Científicas y Técnicas de Argentina, Godoy Cruz 2290, Buenos Aires 1425, Argentina

**Keywords:** Microcystins, [D-Leu^1^]MC-LR, Oxidative stress, Antioxidant capacity, Rat brain

## Abstract

Cyanotoxins are a significant concern due to their frequent presence in Southamerica waters. While numerous studies have investigated the toxic effects of MC-LR, knowledge regarding the toxicity of [D-Leu^1^]MC-LR remains limited. The aim of this study was to determine the impact of [D-Leu^1^]MC-LR and MC-LR administration on different brain structures in rats and the resulting modifications in oxidative stress. Male Sprague-Dawley rats were divided into two groups and administered 5 intraperitoneal injections of mixed MCs at doses of 2 and 15 µg kg^−1^ for each injection over a 21-day period, *i.e.* total doses of 10 and 75 µg kg^−1^.The MCs consisted on MC-LR (3 %), [D-Leu^1^]MC-LR (96.7 %) and others (0.3 %) isoforms. To evaluate the effect of treatments with different doses, the concentration of both MC isoforms, reactive oxygen species (ROS), lipid damage and antioxidant activity were measured in the cerebral cortex, hippocampus, striatum and cerebellum. The results revealed variability in MC concentration across brain regions. The accumulation rate of MC-LR was 2000 times higher than that of [D-Leu^1^]MC-LR, regardless of the dose administered at different areas. Taken together, our results highlighted that chronic exposure to MCs induced a mild oxidative stress in the rat brain characterized by increased ROS and antioxidant defense activation due to [D-Leu^1^]MC-LR in both the striatum and cortex at high dose. At low dose, the uptake of only MC-LR was determined in the cerebellum and hippocampus, resulting in increased ROS levels but no change in CAT activity in the hippocampus. In contrast, in the cerebellum, there was a decrease in ROS, possibly due to increased CAT consumption. However, the absence of detection of an MC variant under certain conditions does not allow for the exclusion of its metabolic effects. Chronic MC administration resulted in dose- and region-dependent distribution within the rat brain. Reactive species levels and cellular responses also varied by dose and region.

## Highlights

• Injected MC isoform concentration varied by dose and brain region.
• At 10 and 75 μg kg^-1^, the MC-LR accumulation rate was higher than the [D-Leu^1^]MC-LR.
• Chronic MCs exposure caused dose/area-dependent mild oxidative stress (↑ROS, CAT activation).
• With higher MC dose all brain regions exhibited elevated reactive species.

## Graphical abstract

**Figure:**
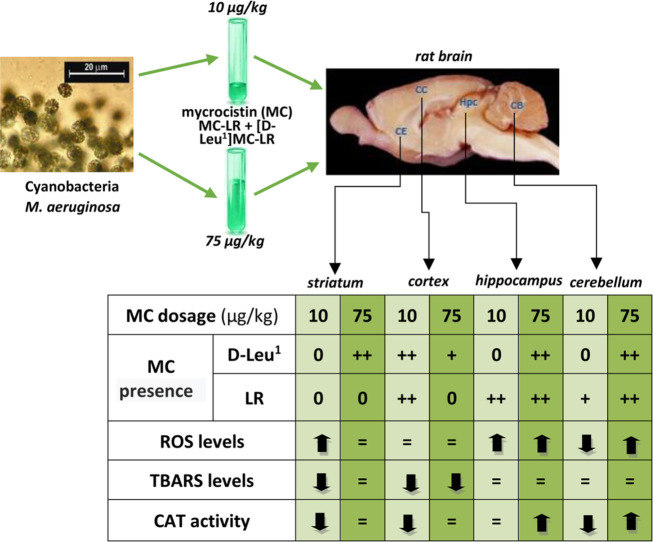

## 1 Introduction

Human poisoning has been reported worldwide due to consumption of water contaminated with toxins produced by toxic strains of cyanobacteria (U.S. EPA, 2015). These cyanotoxins are classified based on their mechanism of action and impact on mammals (Sivonen and Jones, 1999). When cyanotoxins like microcystins (MCs) contaminate water sources used for drinking, they pose a significant threat to human health. MCs are of global concern not only due to their ability to cause acute poisoning but also because of their potential to promote cancer through chronic exposure (Svirčev et al., 2009; Drobac *et al*., 2013).

*Microcystis aeruginosa* blooms are expanding worldwide mainly due to the eutrophication of ponds, lakes, and rivers, often in conjunction with increased temperatures, and have major environmental and human and animal health impacts (Harke *et al*., 2016). *M. aeruginosa* is also widely distributed in freshwater bodies in Argentina (Giannuzzi *et al*., 2011) and is a producer of MCs, with MC-LR being the most toxic variant. Reports on massive proliferations of *Microcystis* sp. in the Río de la Plata have alarmingly increased by the end of 1990′s, associated with water quality changes due to human activities (*i.e.*, urbanization, agriculture, untreated effluent discharges) (Pizzolon *et al*., 1999). >279 isoforms of MCs have been described (Chen *et al*. 2021a), primarily differing in the l-amino acids at positions 2 and 4, which result in differences in toxicokinetic and toxicodynamic properties (Rinehart *et al*., 1994). These compounds are the most widespread cyanobacteria toxins detected in freshwater (Spoof and Catherine, 2017), and many cyanobacterias, including *Microcystis, Planktothrix, Anabaena, Nostoc, Aphanizomenon, and Anabaenopsis*, among others, are capable of synthesizing them (Bittencourt-Oliveira *et al*., 2014).

MCs require active transport to cross cell membranes. The most well-known mechanism of action of MCs is the inhibition of protein serine/threonine phosphatases, which leads to phosphoprotein deregulation, tumor promotion, and apoptosis (Vichi *et al*., 2016). Inhibition of phosphatases (PP) type 1 and 2A is one of the main events that induce neurotoxicity by MCs (MacKintosh *et al*., 1990). This suggests that significant amounts of MCs could cross the blood-brain barrier and cause brain pathology (Huber *et al*., 2007; Westholm *et al*., 2009).

Although MCs are primarily considered hepatotoxins, they can also damage other organs, such as the intestines, heart, kidneys and stomach (Li *et al*., 2024; Moreno *et al*., 2003; Qiu *et al*., 2009). MCs exhibit significant neurotoxic activity in mammals, causing notable behavioral changes, neuronal loss, and severe morphological alterations. They also induce oxidative stress at the brain level and cause changes in the neuronal cytoskeleton (Meng *et al*., 2011). Additionally, research has suggested memory loss as a potential consequence of MC-LR exposure (Maidana et al., 2006). Histological and ultrastructural injuries, along with serious oxidative damage, have been observed in the rats intrahippocampally injected with MC-LR (10 μg/L for 1 μL) (Li *et al*., 2014), including shrunken pyramidal cell nuclei, cellular edema, and decreased neuronal density. However, the exact mechanism behind the neurotoxicity of MCs remains unknown.

Most studies have focused on MC-LR due to its prevalence and well-documented neurotoxic effects in several experimental models (Hinojosa *et al*., 2019). However, other more toxic congeners, such as MC-LW and MC-LF, have also been investigated (Feurstein *et al*., 2009, 2010). Regarding their effects on the nervous system, Hu *et al*. (2016) and Mello *et al*. (2018) have reviewed the primary mechanisms of MC neurotoxicity at different levels.

Prolonged natural exposure of wild and domestic animals to lower levels of MCs may be undetectable. However, this exposure is significant, as it could become an important route, similar to the natural exposure to MCs potentially present in daily drinking water.

Furthermore, MC-LR can disrupt the antioxidant system, leading to oxidative stress by increasing reactive oxygen species (ROS), which in turn triggers apoptosis (Chen and Xie, 2016; Li et al., 2015a; Liu et al., 2016, 2024; Wang et al., 2013). Oxidative stress arises from an imbalance between ROS and antioxidants, which include both enzymatic antioxidants (*e.g.*, catalase [CAT] and superoxide dismutase [SOD]) and non-enzymatic compounds, such as hydrophilic molecules like GSH and ascorbate (AH−), and lipophilic compounds like α-tocopherol (α-T) (Gonzalez et al., 2015). Excessive ROS production also contributes to toxicity from MC exposure, inducing lipid peroxidation (LPO), reducing GSH and alters malondialdehyde (MDA) concentrations, and affecting the expression and activity of antioxidant enzymes such as CAT, SOD, GSH peroxidase (GPX), GSH reductase (GR), and glutathione-S-transferase (GST) (Huang et al., 2015; Jayaraj et al., 2006; Li et al., 2015b). Additionally, MCs promote ROS production and inhibit PP2A activity, which can lead to DNA alterations (Chen and Xie, 2016).

Therefore, the general objective of this study was to determine the impact of the administration of [D-Leu^1^]MC-LR and MC-LR in various brain structures in rats (cortex, striatum, cerebellum and hippocampus) in terms of the presence of both MC isoforms and oxidative stress and the resulting modifications in antioxidant protection. Additionally, the effect of [D-Leu^1^]MC-LR on antioxidant capacity will be discussed.

## 2 Materials and methods

### 2.1 Cyanobacteria strain and toxins

This study utilized a toxic cell extract from the *M. aeruginosa* wild-type strain CAAT 2005–3, isolated near Pila, Buenos Aires, Argentina (Rosso *et al*., 2014). This strain has been previously identified as a [D-Leu^1^]MC-LR producer (Giannuzzi et al., 2016). HPLC/MS analysis confirmed that the most prevalent microcystin was [D-Leu^1^]MC-LR (96.7 %), a variant with inhibitory effects on phosphatases similar to MC-LR (Sedan *et al*., 2020). Additionally, MC-LR was detected in the extracts but in lower quantities (3 %) (Malanga *et al*., 2019) (**Fig. SM 1 A, B**). Cultures were grown in liquid BG-11 medium (Rippka *et al*., 1979) under a 14/10 h light/dark photocycle at 26 °C with a light intensity of 30 μE m^−2^ s^−1^, monitored daily with an IL spectroradiometer.

[D-Leu^1^]MC-LR and MC-LR from the strain were purified using established methods (Barco *et al*., 2002) with slight modifications. Cells were first lysed by three freeze-thaw cycles, followed by additional cell breakage via sonication (Omni-Ruptor 400, 15 min). MCs were then extracted with chloroform/methanol (50/50; v/v). The extract was centrifuged at 7000 rpm for 8 min, and the supernatant was applied to a C18 cartridge, preconditioned by washing with 100 % methanol and distilled water. The cartridge was subsequently washed with water, and MCs were eluted with methanol. The aqueous fraction was concentrated using a rotavapor (Decalab, R-23, Buenos Aires, Argentina).

Finally, MC separation (50 μL sample injection volume) was performed by reverse-phase chromatography on a C18 column. The analytical column (150 × 4.6 mm) was packed with 5 μm particles (Thermo) and maintained at 25 °C. The flow rate was 1 mL min^−1^, with a flow division before entering the ESI (0.2 mL min^−1^ to the ESI-MS). Gradient elution was performed with two eluents: eluent A (water) and eluent B (acetonitrile), both containing 12.7 mM formic acid. The initial conditions were 30 % B, followed by a linear gradient to 70 % B over 12 min, a 3-min isocratic elution with 70 % B, and a linear gradient to 30 % B over 5 min (total run time: 20 min). Toxin quantification was conducted using the HPLC-MS method (Barco *et al*., 2002). The concentration and identification of MC-LR were performed by comparison with a commercial standard (Sigma Chemical Co., St. Louis, MO, USA). The [D-Leu^1^]MC-LR was purified using HPLC-UV, employing the same injection volume, mobile phase and gradient conditions as previously described. Detection was carried out using a UV detector set at a fixed wavelength of 238 nm. The peak corresponding to [D-Leu^1^]MC-LR or MC-LR was collected separately and concentrated with a previously activated C18 cartridge. Pure [D-Leu^1^]MC-LR and MC-LR were eluted with a solution of methanol:water (90:10, v:v) after which the methanol was evaporated. The total concentration of [D-Leu^1^]MC-LR obtained after purification was 6 ppm. The resulting extracts were preserved at −20 °C until use. The concentration of standard MC-LR from Sigma was 10 ppm. The initial concentration of the MC mixture, extracted from *M. aeruginosa* cultures and used to prepare the solutions for the time-course injections at both experimental doses, was 60 ppm.

### 2.2 Animals and treatments

Two independent experiments were conducted, with injections of 10 and 75 µg kg⁻¹ body weight (BW) of MCs, respectively (both final doses). In each experiment, twelve male *Sprague Dawley* rats, weighing between 220 and 245 *g*, were acclimated for 2 weeks at 22±2 °C, with food and water available *ad libitum*, and a light-dark cycle of 14:10 h (light ON: 7:00 a.m.) before their injections. The rats utilized in the experiments were aged 9 and 10 weeks for the cohorts exposed to 10 and 75 µg kg⁻¹ BW, respectively (Table 1). The rats were randomly divided into 2 groups of 6 animals each one, and placed in special cages. Thus there was the control group (CG), consisting of animals injected with saline solution without exogenous MCs; and (2) the MC group (MC), consisting of rats injected with exogenous MCs. In MC group rats was injected with increasing intraperitoneal (i.p.) doses of [D-Leu^1^]MC-LR and MC-LR being total MC doses of 10 and 75 µg kg⁻¹ BW (see above). The relative proportions of MC-LR and [D-Leu^1^]MC-LR in the injected mixture were 3 % and 96.7 %, respectively.

**Table 1:** Body weights, brain weights and brain/body weight ratios in both experimental groups at the end of study. Mean and standard deviation are reported for treatment and control rats exposed to MC at 10 and 75 µg kg-1. Within each parameter, treatment and control means denoted by the same letter showed no statistically significant differences (ANOVA, *p* > 0.05).

Treatment	Age (wk)	Initial Body	Final Body	% increment	Brain	Ratio Brain/Body
		Weights (g)	Weights (g)	Body Weight	Weights (g)	Weight
**(A) MC 10µg Kg^-1^**						
1	9	229	305	33,19	1,65	0,54
2	9	223	300	34,53	1,62	0,54
3	9	220	300	36,36	1,63	0,54
4	9	219	301	37,44	1,68	0,56
5	9	225	308	36,89	1,67	0,54
6	9	227	310	36,56	1,69	0,55
Average ± SD		(**a**)223,8 ± 3,9	(**a**)304±4,3	(**a**)35,8 ± 1,6	(**a**)1,66±0,05	(**a**)0,54±0006
**Control**						
1	9	225	306	36,00	1,66	0,54
2	9	217	304	40,09	1,63	0,54
3	9	231	312	35,06	1,71	0,55
4	9	228	311	36,40	1,7	0,55
5	9	226	308	36,28	1,69	0,55
6	9	224	309	37,95	1,68	0,54
Average ± SD		(**a**)225,2 ± 4,7	(**a**)308,3 ± 3	(**a**)36,9 ± 1,8	(**a**)1,68±0,05	(**a**)0,54±0005
**(B) MC 75µg Kg^-1^**						
1	10	239	310	29,71	1,7	0,55
2	10	241	322	33,61	1,81	0,56
3	10	235	315	34,04	1,75	0,56
4	10	247	335	35,63	1,83	0,55
5	10	244	320	31,15	1,74	0,54
6	10	248	331	33,47	1,84	0,56
Average ± SD		(**x**)242,3 ± 4.9	(**x**)322,2 ± 9.5	(**x**)32,9 ± 2.1	(**x**)1,8 ± 0.08	(**x**)0,5 ± 0.01
**Control**						
1	10	238	318	33,61	1,72	0,54
2	10	248	336	35,48	1,82	0,54
3	10	241	325	34,85	1,79	0,55
4	10	243	323	32,92	1,74	0,54
5	10	250	340	36,00	1,9	0,56
6	10	252	334	32,54	1,79	0,54
Average ± SD		(**x**)245,3 ± 5,5	(**x**)329,3 ± 8,7	(**x**)34,2 ± 1,4	(**x**)1,8 ± 0,06	(**x**)0,5 ± 0,01

Rats received MC extract via i.p. injection every four days for a total of twenty-one days (**Tabla SM 1**). Two doses were used: a low daily dose of 2 µg MCs kg⁻¹ BW and a high daily dose of 15 µg MCs kg⁻¹ BW body weight. The toxin was freshly prepared by diluting a stock solution with saline (0.9 % w/v). Control groups received an equivalent volume of saline solution via the same route. Following treatment, rats from both the treated and control groups were euthanized with CO₂ in their home cages to minimize stress, and brain samples were collected.

The brains were dissected, washed in a saline solution at 4 °C and separated. Brain dissection was performed according to Czerniczyniec *et al*. (2011). Under a stereoscopic magnifying glass (Zeiss) and on an ice bed, the cerebral cortex, hippocampus, striatum, and cerebellum were dissected. These tissues were rapidly removed, immediately frozen, and stored at −80 °C until further determinations, which were conducted within one to two months. Samples were evaluated for reactive species, enzymatic antioxidants like catalase (CAT), lipid damage, and toxin concentration (see above).

The experimental protocol was approved by the Institutional Animal Care and Use Committee (IACUC) of the University of Morón. Experimental animals were treated according to the code of ethics proposed by the Canadian Council on Animal Care, as well as by Argentinean laws.

### 2.3 Extraction and determination of MCs on brain areas

Approximately 0.1 *g* of frozen samples from each brain area were gently sonicated for 1 min and extracted using 2 mL of 0.01 M EDTA solution in 5 % acetic acid. The suspensions were then centrifuged at 10,000 rpm for 8 min, and each supernatant was directly concentrated on an SPE cartridge (C18, 5 g), which had been preconditioned by washing with 50 mL of 100 % MeOH and 50 mL of distilled water. The cartridge was subsequently washed with 50 mL of distilled water, followed by 100 mL of 20 % methanol. Elution from the cartridge with 100 mL of 90 % methanol was used for the qualitative and quantitative analysis of MCs by HPLC–MS (as described in the Toxins section) (**Fig. SM 2 A, B**).

**Fig. 1:**
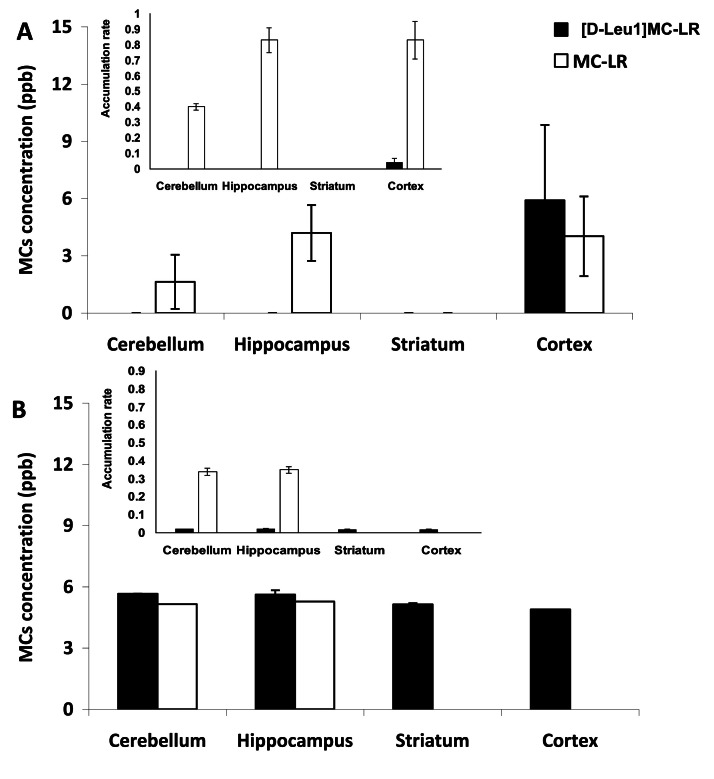
MCs concentration (ppb) in the different brain regions analyzed (cerebellum, hippocampus, striatum, cortex) after i.p. injection of (**A**) 10 µg kg^−1^ and (**B**) 75 µg kg^−1^ body weight. The mean of each MCs isoform (*n* = 6) is represented with its standard deviation. Black and white bars represents to [D-Leu^1^]MC-LR and MC-LR, respectively. Insert: accumulation rate for each MCs in different areas at both doses, the mean and standard deviation.

The accumulation rate of MCs in each brain region and for each dose was calculated by summing the amount of toxin administered over each injection day (**Table SM 1**) and the corresponding values obtained by HPLC/MS from tissues (Fig. 1**A, B insert**). The relative proportions of MC-LR and [D-Leu^1^]MC-LR in the injected mixture were 3 % (1.8 ppm) and 96.7 % (58.02 ppm), respectively, providing a basis for comparing their accumulation rates.

### 2.4 Oxidation of 2´7´-dichlorodihidrofluorescein diacetate (DCFH-DA)

Intracellular 2,7-dichlorofluorescin (H_2_DCF) oxidation is often used to measure the production of reactive species within cells (McDowell *et al*., 2013). The oxidation of DCFH-DA was assessed in brain homogenates, following the method of Malanga *et al*. (1997). Tissue from each brain area (0.05 *g*) was homogenized in 100 mM Tris–HCl (pH 7.4) with 2 mM EDTA and 5 mM MgCl₂. The homogenates were then incubated with 980 µL of 30 mM HEPES (pH 7.2), 200 mM KCl, 1 mM MgCl₂, and 10 µL of DCFH-DA (1 mg/mL) diluted in methanol. Samples were incubated for 30 min at 37 °C, and fluorescence was determined at λex = 488 nm and λem = 525 nm.

### 2.5 TBARS content

Cellular TBARS content was used as an indicator of lipid peroxidation. TBARS content allows for a rough estimate of the presence of aldehydes, although most reactivity originates from malondialdehyde (MDA). MDA is a product of lipid peroxidation that can react with thiobarbituric acid under acidic and hot conditions to allow a colorimetric assay (Janknegt *et al*., 2008). Thus, TBARS content was measured using a modified fluorescence method (Boveris and Puntarulo, 1998). An aliquot (0.05 *g*) of brain samples was homogenized with 1 mL of 40 mM potassium phosphate buffer and 120 mM KCl (pH 7.4). To 0.1 mL of the homogenate, 0.05 mL of 4 % (w/v) butylated hydroxytoluene (BHT) and 0.25 mL of 3 % sodium dodecyl sulfate were added. After mixing, 1 mL of 0.1 N HCl, 0.15 mL of 10 % (w/v) phosphotungstic acid, and 0.5 mL of 0.7 % (w/v) 2-thiobarbituric acid were added. The mixture was heated for 45 min in boiling water, and TBARS were extracted into 1 mL of n-butanol. After brief centrifugation, the fluorescence of the butanol layer was measured at λex = 515 nm and λem = 555 nm. Malondialdehyde standards were prepared from 1,1,3,3-tetramethoxypropane.

### 2.6 Catalase activity assays

Tissue samples (0.05 *g*) were homogenized in 40 mM potassium phosphate buffer and 120 mM KCl (pH 7.4), then centrifuged at 10,000 rpm for 10 min. The resulting supernatant was used to measure CAT activity spectrophotometrically by monitoring the decomposition of H₂O₂ at *k* = 240 nm. The reaction mixture consisted of 50 mM potassium phosphate buffer (pH 7.0) containing 10 mM H₂O₂ (Aebi, 1984).

### 2.7 Statistical analyses

One-way ANOVA was performed using Statistica (version 9) to determine the significance of differences observed between treatments for each parameter value. Normality was verified using a Kolmogorov–Smirnov test. A Tukey test was additionally performed to identify significant differences between treatments (Scheiner, 2001).

## 3 Results

An average 36 % and 33 % increase was registered by comparing the weight of the rats before and after the MC injection (10 and 75 µg kg⁻¹ BW, respectively) with no significant differences between them (*p* > 0.05). The same trend was observed for the control rats (Table 1). The brain/body weight ratio at the end of the experiment was between 1.7 and 1.8. No significant differences were found between treated and control rats for both doses evaluated (*p* > 0.05, Table 1).

The MCs concentration and isoform varied in each studied brain region and were dose-dependent. For the 10 µg kg⁻¹ BW dose, no MCs were detected in the striatum, and only a very low concentration of MC-LR was found in the cerebellum. This isoform was also detected in the hippocampus and cortex, with higher concentrations observed in the cortex compared to the cerebellum. In the cortex, the [D-Leu^1^]MC-LR isoform was also detected, with a concentration similar to that of MC-LR (Fig. 1**A**). When rats were injected with 75 µg kg⁻¹ BW of MCs, the [D-Leu^1^]MC-LR variant was detected in all brain areas at similar concentrations. Contrary to the results obtained with the low dose, MC-LR was not detected in the cortex at this high dose. However, MC-LR was detected in the hippocampus, at a concentration similar to that observed with the low dose, and in the cerebellum, where the concentration was higher than that recorded at 10 µg kg⁻¹ BW (Fig. 1**B**).

The MCs accumulation rate was 50 % lower at the high compared to the low dose for MC-LR in hippocampus and cerebellum where it was almost the same value at high than in low MC doses. For [D-Leu^1^]MC-LR the accumulation rate was a 50 % lower in cortex at high compared with low MC doses. However, the accumulation rate of MC-LR was 2000 times higher than that of [D-Leu^1^]MC-LR, regardless of the dose administered at different areas (Fig. 1 **insert A, B**).

**Fig. 2:**
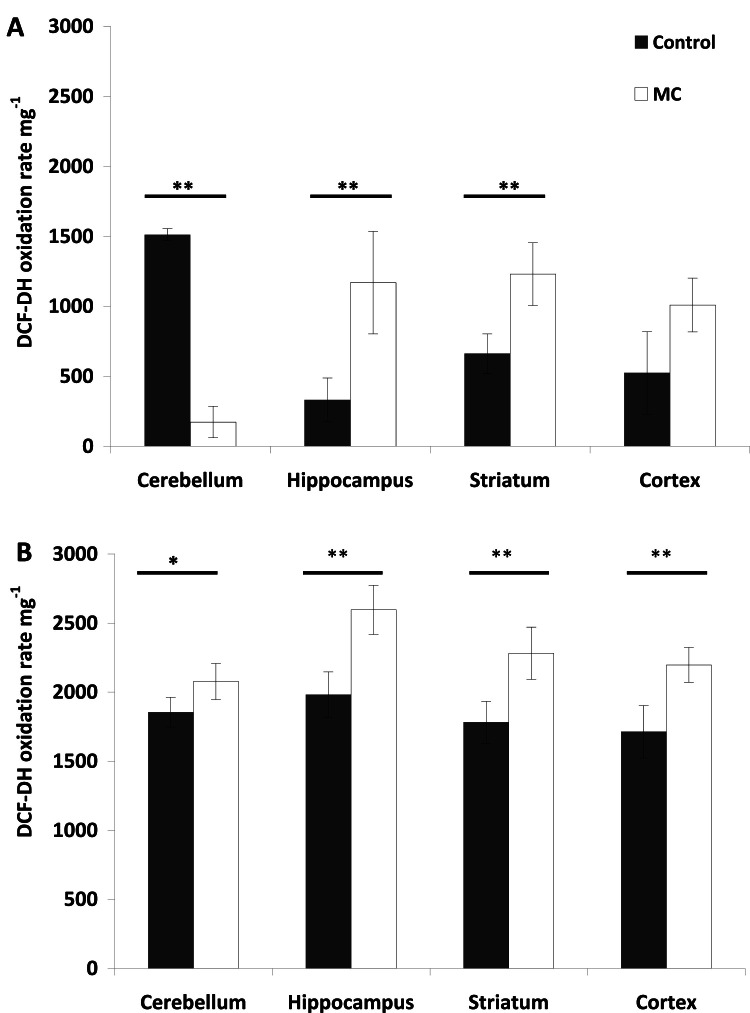
DCF-DA oxidation rate (AU *h*^−1^ per mg^−1^) in the different brain regions analyzed (cerebellum, hippocampus, striatum, cortex) after i.p. injection of (**A**) 10 µg kg^−1^ and (**B**) 75 µg kg^−1^ body weight. The mean (*n* = 6) is represented with its standard deviation. Significant differences respect to control brain (black bars) are shown with *P* < 0.05 (*) and *P* < 0.01 (**) (Tukey test).

**Fig. 3:**
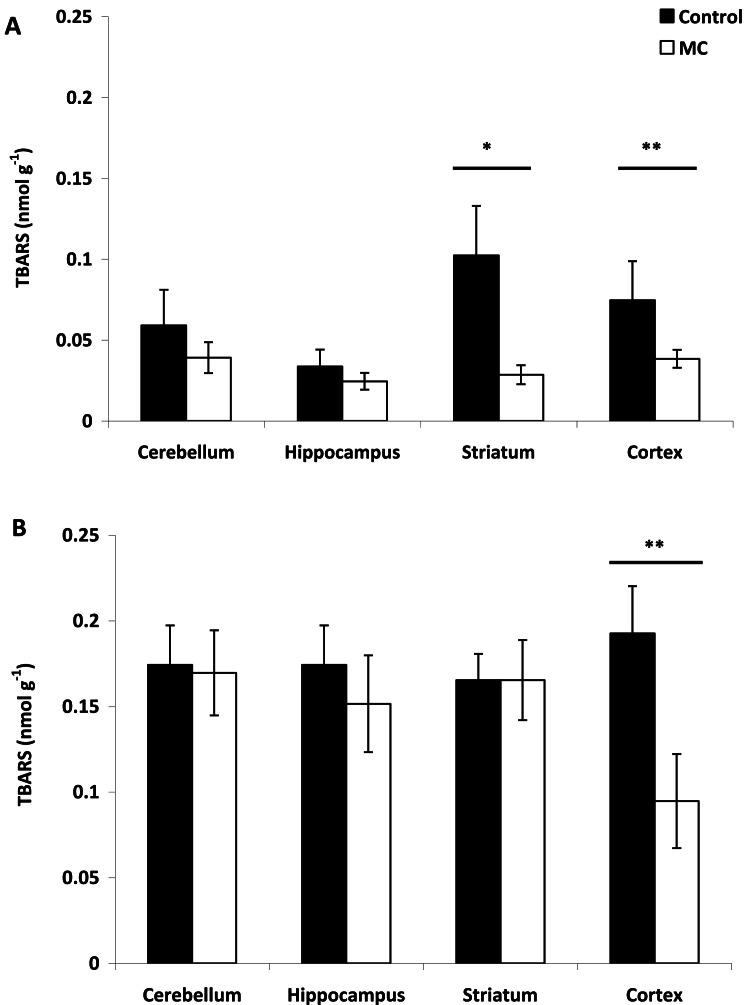
TBARS content (nmol *g*^−1^) in the different brain regions analyzed (cerebellum, hippocampus, striatum, cortex) after i.p. injection of (**A**) 10 µg kg^−1^ and (**B**) 75 µg kg^−1^ body weight. The mean (*n* = 6) is represented with its standard deviation. Significant differences respect to control brain (black bars) are shown with *P* < 0.05 (*) and *P* < 0.01 (**) (Tukey test).

**Fig. 4:**
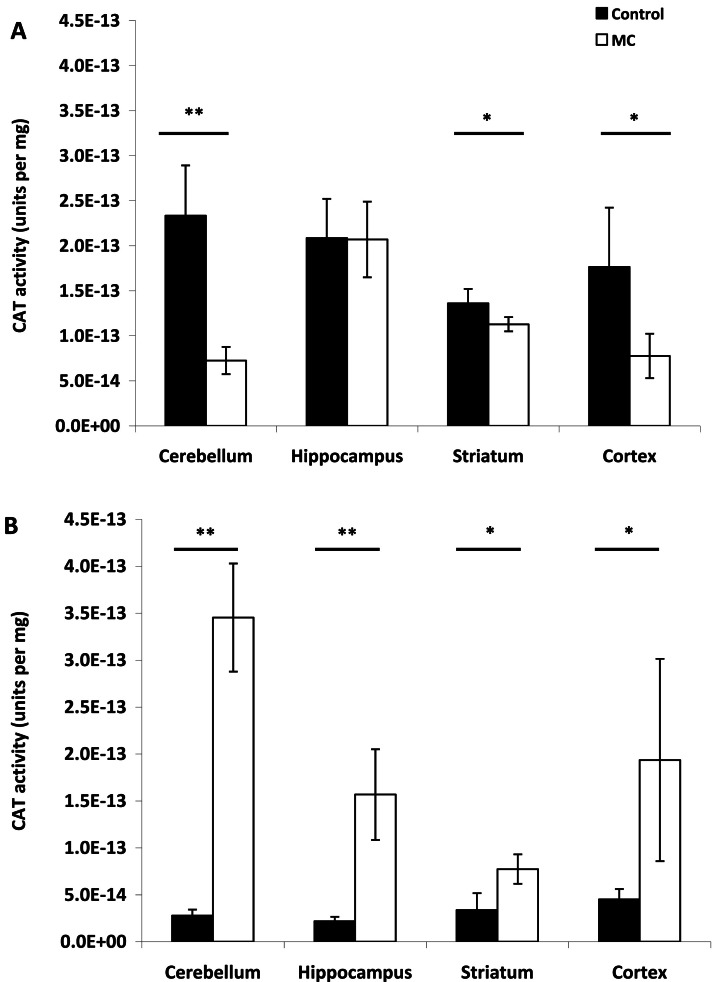
CAT brain enzymatic antioxidant activity (Units per mg) in the different brain regions analyzed (cerebellum, hippocampus, striatum, cortex) after i.p. injection of (**A**) 10 µg kg^−1^ and (**B**) 75 µg kg^−1^ body weight. The mean (*n* = 6) is represented with its standard deviation. Significant differences respect to control brain (black bars) are shown with *P* < 0.05 (*) and *P* < 0.01 (**) (Tukey test).

The significant difference in control values for stress parameters across experimental doses (Fig. 2, Fig. 3, Fig. 4) may be due to the experiments being conducted a few months apart, despite both experimental groups originating from the same Animal Facility. The values for each parameter are coherent when considering the relationship between the level of reactive species, lipid peroxidation, and CAT, which is a result of variability between experimental groups. The animals used in this study were all of the same strain, age, and with similar average weights (Table 1). The production of reactive species was dose-dependent and varied across the different brain regions analyzed. Following injection with 10 µg kg⁻¹ BW, a significant increase in reactive species was observed in both the hippocampus (*p* < 0.05) and striatum (*p* < 0.01), with no significant differences compared to the control in the cortex (*p* > 0.05). In the cerebellum, a lower concentration of reactive species was detected in treated rats compared to the control (*p* < 0.01) (Fig. 2**A**). In contrast, injection with 75 µg kg⁻¹ BW resulted in a significant increase in reactive species in all brain regions analyzed compared to the control group (*p* < 0.01) (Fig. 2**B**).

At a low dose of MCs, lipid damage was observed in both the striatum (*p* < 0.01) and cortex (*p* < 0.05) compared to TBARS values in control brain rats. No significant differences were found in TBARS levels in the cerebellum and hippocampus between treated and control brain rats (*p* > 0.05) (Fig. 3**A**). In contrast to the observations at low MCs doses, injection with 75 µg kg⁻¹ BW MCs did not induce lipid damage in any of the analyzed brain regions, as TBARS content did not show significant differences between treated and control rats (*p* > 0.05). Notably, TBARS values were significantly lower in the cortex of treated rats compared to controls (*p* < 0.01) (Fig. 3**B**).

Regarding CAT activity, no significant differences were observed in the hippocampus between treated rats and controls following injection with 10 µg kg⁻¹ BW (*p* > 0.05). In the cerebellum (*p* < 0.01), striatum (*p* < 0.05), and cortex (*p* < 0.05), catalase activity was significantly lower in treated rats compared to controls (Fig. 4**A**). For high doses of MCs, CAT activity was significantly higher in all analyzed brain regions compared to controls (Fig. 4**B**).

## 4 Discussion

Studies have shown that MCs affect human health mainly through contaminated water and food, inhalation, body contact, dietary supplements, and hemodialysis (Alosman *et al*., 2020; Massey *et al*., 2018, 2020; Pouria *et al*., 1998). LD_50_ values of 36–250 µg kg⁻¹ BW have been reported for MC-LR via i.p., i.v., intranasal, or inhalation routes, depending on the strains used in mice and rats (Creasia, 1990; Dawson, 1998; Fitzgeorge *et al*., 1994). Chen et al. (2016) reported a MC-LR LD_50_ values of 82.7 µg kg^−1^ BW on male *Sprague-Dawley* rats after 24 h i.p. injection.

Microcystins are readily absorbed into the bloodstream after ingestion and are rapidly distributed throughout the body, particularly to well-perfused organs like the liver, intestines, kidneys, lungs, and brain (Cazenave *et al*., 2005; Meng *et al*., 2011). Interestingly, in our study, no MCs were detected in the striatum, and only MC-LR was found in the cerebellum and hippocampus following intraperitoneal (i.p.) administration of 10 µg kg⁻¹ BW to rats. The neurotoxic potential of different MC variants depends on the expression and functionality of transport systems in the blood-brain barrier (Feurstein *et al*., 2010). It has been demonstrated that MCs need organic anion-transporting polypeptides (OATPs in humans/Oatps in rodents) to cross cell membranes (Chen and Xie, 2016). Besides the liver, the organic transporter OATP1b2 is also present in the brain (Fischer *et al*., 2005). The expression of five MC-specific OATPs in the brain of murine rodents has been identified, indicating their involvement in the neuronal uptake of MCs (Feurstein *et al*., 2009, 2010).

At low dose only MC-LR of the MCs mixture was detected in the cerebellum and hippocampus, resulting in increased reactive species levels but no change in CAT activity in the hippocampus. The low MC concentration in the cerebellum likely did not generate reactive species that cause lipid peroxidation, possibly due to increased CAT consumption. However, lack of detection of an MC variant in certain conditions doesn't mean that its metabolic effects are absent. Oxidative stress is typically defined as an adverse reaction resulting from the exposure of molecules, cells, or tissues to excessive levels of oxidants, particularly ROS (Ding and Ong, 2003). Under such condition, cells or tissues can be damage by increasing lipid peroxidation. Several reports have documented MC-induced lipid damage in rat and mouse cells (Ding *et al*., 2003; Weng *et al*., 2007). Acute MC exposure has been shown to cause significant oxidative stress in various mammalian organs (Guzman and Solter, 1999; Moreno *et al*., 2005). An increase in ROS levels beyond their normal range, leading to cellular dysfunction, is termed oxidative stress. ROS are typically mitigated by the activity of antioxidant enzymes such as catalase (CAT) and superoxide dismutase (SOD) (Gonzalez *et al*., 2015; Packer, 1995). As long as these antioxidant defenses are not overwhelmed, they can restore ROS levels to their normal state (Lushchak, 2014). When the activity of cellular antioxidant defense system decreases or the production of ROS increases, oxidative stress may occur (Jayaraj *et al*., 2006). Therefore, oxidative stress, caused by the increase in intracellular ROS, plays a major role in the toxic effects of MCs and could induce an antioxidant response in exposed cells and tissues (Ding *et al*., 2001; Gonzalez *et al*., 2015). In the present study there was a significantly increase in reactive species in both experimental doses with a significant increase in CAT activity at high dose. These results demonstrate that MC injection induced a mild oxidative stress condition (Halliwell, 2006), despite the absence of lipid peroxidation at both experimental doses. The induction of enzymatic antioxidant defenses after MCs exposure represents an adaptive mechanism that enables cells to overcome oxidative stress and promote survival by preventing apoptosis.

Oxidative stress has also been linked to neurotoxic effects in fish and other animals, including cognitive impairment in humans. In the present study, the absence of lipid damage at low MC doses could be explained by the low CAT activity observed under these conditions. Despite the low dose administered and the absence of any MC isoforms in the striatum, it is notable that a significant increase in reactive species production did not result in lipid peroxidation or CAT activation. Such results could be explained by the potential consumption of MCs, whose antioxidant function has recently been demonstrated (Malanga *et al*., 2019). Malanga *et al*. (2019) showed that MC-LR efficiently scavenged ascorbate and hydroxyl radicals (hydrosoluble radicals) *in vitro* via [D-Leu^1^]MC-LR addition. This aligns with the established role of MCs in mitigating oxidative stress (Van de Waal *et al*., 2011; Zilliges *et al*., 2011). Zilliges *et al*. (2011) observed increased interactions between MCs and cellular proteins under high light and stress conditions in *Microcystis* sp. Nian Wei *et al*. (2016) identified additional MC-bound proteins not reported by Zilliges *et al*., suggesting diverse functions for MCs. In addition, Sevilla *et al*. (2008) reported increased MC production under similar stress conditions, further supporting an intracellular role for MCs in stress mitigation. The increased MC-protein binding under stress conditions (Zilliges *et al*., 2011) might explain the different growth patterns observed between wild-type and MC-deficient cyanobacteria (Zilliges *et al*., 2011; Meissner *et al*., 2015). Thus, the decrease in MC concentration observed in the present study at low doses could be due to MC consumption or protein binding. Regardless of the mechanism, the overall consequence seems to be the avoidance of stress, as previously hypothesized.

Despite the results observed in the cerebellum at low MC doses, higher concentrations of MC-LR were recorded in the hippocampus and cortex. Additionally, the presence of [D-Leu^1^]MC-LR was only detected in the cortex at a similar concentration to that of MC-LR. Fischer *et al*. (2010) demonstrated a differential affinity of the OATP transporters for specific MC isoforms. Thus, MC-LF is taken up more rapidly or efficiently by OATP1B family members than are other MC isoforms. It suggest that MC-LF was transported more efficiently into the cell (toxicokinetics) and thus reached neuronal cells more quickly than did others cianotoxins. Feurstain et al. (2010) also demonstrated that the uptake and subsequent neurotoxicity are MC congener dependent. Thus, these findings could explain the observed differences in isoform distribution across different brain areas in the present study. Furthermore, even if an MC variant isn't detected under specific conditions, its metabolic effects cannot be excluded. While the presence of transporters in the blood-brain barrier that facilitate the entry of both MC-LR and [D-Leu^1^]MC-LR is evident, there may be a more efficient transport mechanism for MC-LR at both doses considering that the accumulation rates were 2000 times higher compared with those for [D-Leu^1^]MC-LR. At high dose the accumulation rate was 50 % lower for MC-LR for hippocampus and 100 % in cortex than at low dose. However at high dose in cortex such rate for [D-Leu^1^]MC-LR was 50 % lower and was present in striatum and cerebellum contrasting with low dose. This observed change in accumulation rate comparing low and high dose, may be attributed to the use of these MCs as scavengers of ROS (see above).

MCs can trigger the production of harmful molecules such as superoxide radicals and hydrogen peroxide (Ding *et al*., 1998). These molecules can damage cell membranes through lipid peroxidation (Ding *et al*., 1998). The elevated MCs concentrations may have contributed to the increased levels of reactive species, even though lipid peroxidation was not observed in either of these areas. This suggests a high level of protection, which was not due to CAT activation as no differences were observed compared to control rats, and in addition, significantly lower CAT activity was recorded in the cortex. Thus, this protection may have been due to the activation of other antioxidant enzymes or compounds not determined in our study. Li *et al*. (2011, 2012) demonstrated that significantly elevated activities of SOD and glutathione peroxidase following intrahippocampal injection of MC-LR were concomitant with increased lipid damage, indicating a rapid response of antioxidant enzymes to oxidative stress, as will be shown in the present study at high MC doses for CAT activity (see above). Additionally, Maidana et al. (2006) demonstrated observed higher GST activity after exposure to the low dose compared to the high after intrahippocampal infusions of MC solutions. Consequently, these antioxidant defense mechanisms demostrated in the previous studies may have also been activated in the present study to prevent lipid damage at low MCs dose.

Different results were observed following the injection of a high MCs dose (75 μg.Kg^−1^). Thus, [D-Leu^1^]MC-LR was detected in all analyzed brain areas, contrasting with observations at lower doses where this isoform was only detected in the cortex (see above). This increase in MCs across the four analyzed areas resulted in a significant rise in reactive species, including in the cerebellum, where no MCs isoform had been detected at the lower dose. Similar to what was observed in the cortex and striatum at a lower MCs dose, no lipid peroxidation was observed in any of the four areas; in fact, lipid peroxidation was significantly lower in the cerebral cortex compared to control rats. Unlike the results at the low dose, CAT activity was significantly higher in each area compared to the control, which may explain the absence of lipid damage. The exclusive presence of [D-Leu^1^]MC-LR at high doses resulted in a significant increase in reactive species and a significant increase in CAT activity in both the striatum and cortex. However, despite only one isoform was detected in some tissues, the effects cannot be attributed only to such isoform due the metabolic processes of the undetermined MC variant could not be demonstrated in this study. Thus, the absence of detection of an MC variant under certain conditions does not allow for the exclusion of its metabolic effects.

Xiong *et al*. (2010), using a similar high MC-LR dose via i.p. injection, reported a significant decrease in the expression of the antioxidant enzyme CAT in rat liver and testis. However, Jayaraj *et al*. (2006) found no change in CAT gene expression in mice administered an i.p. dose of MC-LR at 38 µg kg^−1^ BW. Interestingly, these authors, injectating a similar dose to the one used in the present study but in mice, found decreased CAT activity. These discrepancies highlight the potential influence of several factors on the response of antioxidant enzymes to MCs, including MC dose, MC composition, species, and differences among tissues within the same animal.

While pathological damage within different brain regions can be considered a potential consequence of MC exposure, the present study did not assess such damage. Li *et al*. (2012) demonstrated histological and ultrastructural injury in the hippocampus following MC-LR exposure in a dose-dependent manner, with more severe damage detected in the high-dose group. Chen *et al*. (2021b) reported histopathological damage to the hypothalamus, pituitary, adrenal, ovary, and thyroid in female Sprague-Dawley rats acutely exposed to MC-LR at concentrations similar to those used in our study. The findings of the present study provide a comprehensive understanding of the endocrine-disrupting effects of MCs.

In summary, several studies suggest that MCs may potentially reach the brain. Wang et al. (2008) detected MCs in rat brains, suggesting the possibility of direct neurotoxic effects in both rats and humans. While Falconer et al. (1988) observed minimal MC accumulation in female rats after intravenous administration. Unfortunately, studies in humans suggest a link between oxidative stress and several neurodegenerative disorders, including Alzheimer's and Parkinson's diseases (Giasson *et al*., 2002). The results of this study demonstrate differential uptake of MC-LR and [D-Leu^1^]MC-LR and their specific effects on different brain regions, highlight the complexity of the neurotoxic effects of these compounds. Additionally, it highlight the problems of using only one MC isoform, MC-LR (Grosse et al. 2006), as the basis for MC-related human risk assessment.

## 5 Conclusion

The results of our study demonstrated a clear dose-dependent and region-specific effect of MCs in the brain. Although [D-Leu^1^]MC-LR was the major component of the injected MC mixture, differential accumulation of both isoforms was observed. Notably, MC-LR accumulation was significantly higher than that of [D-Leu^1^]MC-LR across all brain regions and doses. At low dose (10 µg kg^−1^ BW), [D-Leu^1^]MC-LR was observed in the cerebral cortex, and MC-LR was detected in the cerebellum, hippocampus, and cortex following chronic administration of the MCs mixture, but [D-Leu^1^]MC-LR was not detected in the striatum. Both MCs were present in the cerebral cortex. However, at higher doses (75 µg kg^−1^ BW), the concentrations of MCs increased in all areas, with [D-Leu^1^]MC-LR being observed across all regions, and the hippocampus and cerebellum were particularly affected by high concentrations of MC-LR. However, the absence of detection of an MC variant under certain conditions does not allow for the exclusion of its metabolic effects.

To our extent of knowledge, there were only limited studies about the toxic effects of MC on different brain areas of mammals. Taken together, our results highlighted the MCs-induced a mild oxidative stress in the rat brain characterized by increased reactive species and antioxidant defense activation. Interestingly, in the present study, we demonstrate that the access of different MC isoforms to distinct brain regions is also dose-dependent and there is no selective restriction by the blood-brain barrier, as suggested by other studies (Cazenave *et al*., 2005). These findings suggest that the antioxidant response is not uniform across brain regions, indicating that some areas may be less prone to oxidative damage than others. The observed change in MCs accumulation rate comparing low and high dose, may be attributed to the use of these MCs as reactive species scavengers.

## Declaration of competing interest

The authors declare the following financial interests/personal relationships which may be considered as potential competing interests:

Claudio Cervino reports financial support was provided by National Atomic Energy Commission. Marcelo Hernando reports a relationship with National Atomic Energy Commission that includes: employment. If there are other authors, they declare that they have no known competing financial interests or personal relationships that could have appeared to influence the work reported in this paper.

## Data Availability

Data will be made available on request.

## Appendix Supplementary materials

Image, application 1
