# Supplementary material for: Differential impact of microcystins MC-LR and [D-Leu1]MC-LR in different areas of the rat brain after chronic exposure: oxidative stress and antioxidant responses
Source: Curr Res Microb Sci. 2025 May 10;8:100401. doi: 10.1016/j.crmicr.2025.100401 (PMC12143765; doi:10.1016/j.crmicr.2025.100401)
Supplement: Supplementary file 1 [file mmc1.docx]

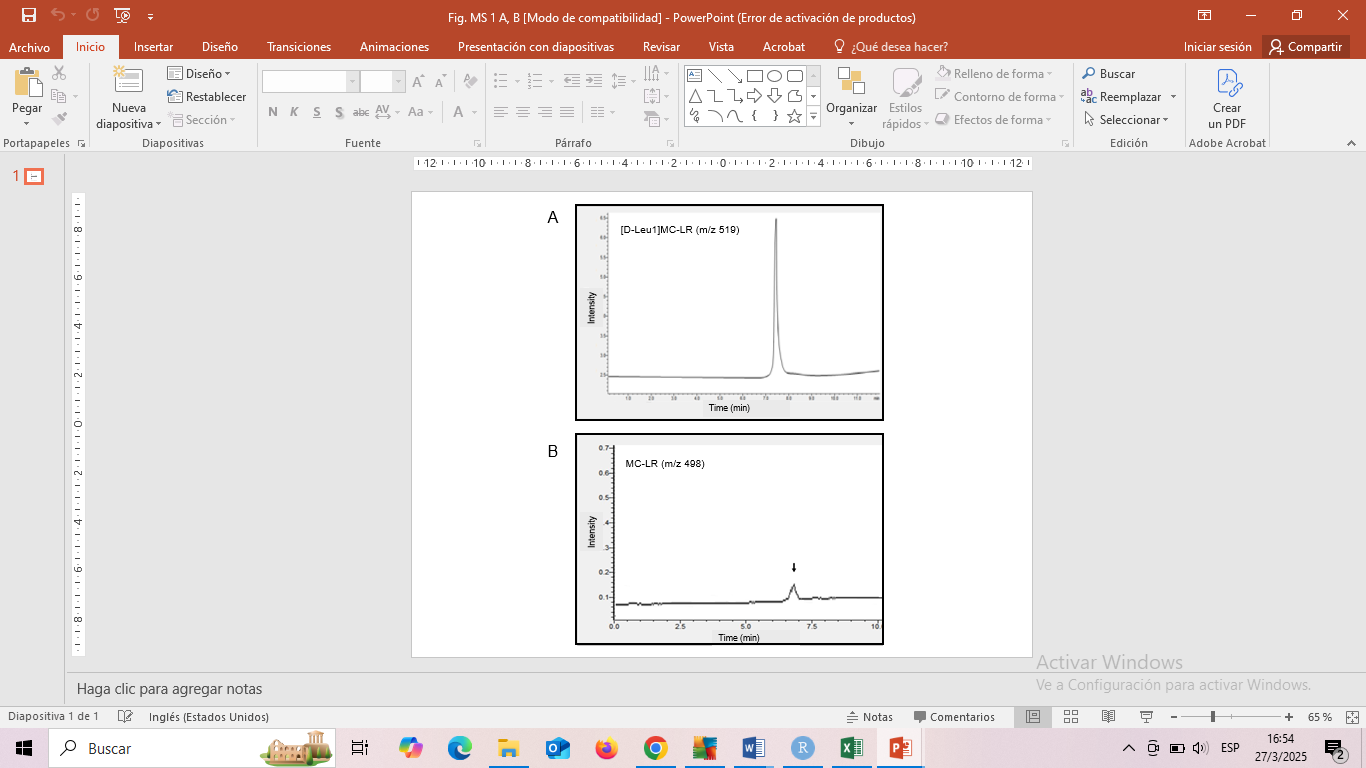


**Figure SM 1**

Ion chromatograms of a solution containing (A) [D-Leu^1^]MC-LR and (B) MC-LR from *M. aeruginosa* extracts.


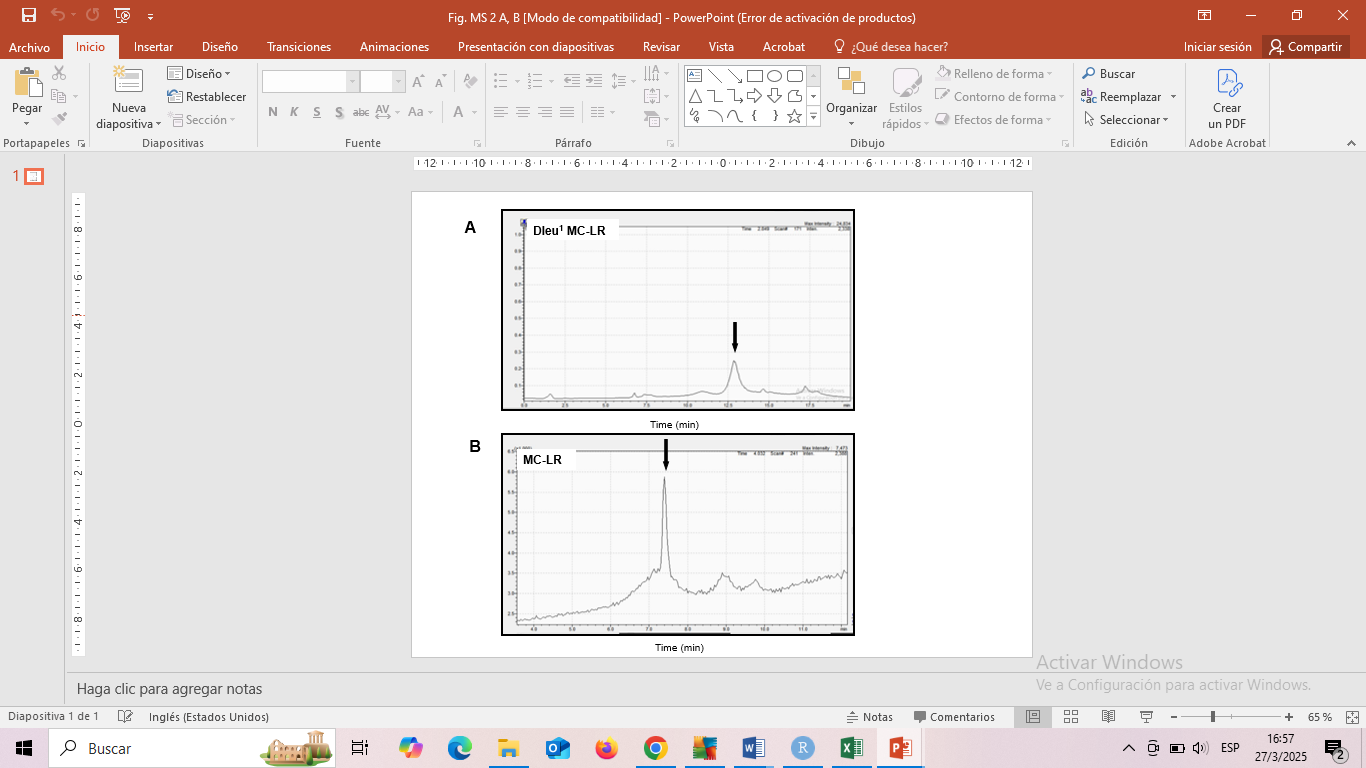


**Figure SM 2**

Ion chromatograms of a solution containing (A) [D-Leu^1^]MC-LR and (B) MC-LR from rat brain areas. The arrow denotes the presence of the indicated MCs.

| **Day of the experiment** | **Injection N^o^** | **Total Dose** | **Amount of toxin injected*** | **Amount of toxin accumulated*** |
| --- | --- | --- | --- | --- |
| **1** | 1 injection | 10 | 2 μg MCs kg⁻¹ BW | 2 μg MCs kg⁻¹ BW |
|  |  | 75 | 15 μg MCs kg⁻¹ BW | 15 μg MCs kg⁻¹ BW |
| **5** | 2 injection | 10 | 2 μg MCs kg⁻¹ BW | 4 μg MCs kg⁻¹ BW |
|  |  | 75 | 15 μg MCs kg⁻¹ BW | 30 μg MCs kg⁻¹ BW |
| **9** | 3 injection | 10 | 2 μg MCs kg⁻¹ BW | 6 μg MCs kg⁻¹ BW |
|  |  | 75 | 15 μg MCs kg⁻¹ BW | 45 μg MCs kg⁻¹ BW |
| **13** | 4 injection | 10 | 2 μg MCs kg⁻¹ BW | 8 μg MCs kg⁻¹ BW |
|  |  | 75 | 15 μg MCs kg⁻¹ BW | 60 μg MCs kg⁻¹ BW |
| **17** | 5 injection | 10 | 2 μg MCs kg⁻¹ BW | 10 μg MCs kg⁻¹ BW *(final dose)* |
|  |  | 75 | 15 μg MCs kg⁻¹ BW | 75 μg MCs kg⁻¹ BW *(final dose)* |
| **22** | Euthanasia | Rapid extraction of the brain in each control group (N= 6) and in each treatment (N= 6) with different doses of microcystin; separation of cerebral cortex, cerebellum, striatum and hippocampus for analysis. | | |

**Table SM 1**

Protocol for daily intraperitoneal administration of MCs at different doses. In each experiment with each dose of 10 and 75 μg kg^-1^, a control group of 6 rats (MC= 0 μg kg⁻¹, in saline solution) and a group of 6 rats treated with the MC doses were used. MC doses consisted of a mixture of (D-leu^1^)MC-LR (96.7%), MC-LR (3.0%), and other isoforms (0.3%).
